# Novel Clade C-I *Clostridium difficile* strains escape diagnostic tests, differ in pathogenicity potential and carry toxins on extrachromosomal elements

**DOI:** 10.1038/s41598-018-32390-6

**Published:** 2018-09-17

**Authors:** Gabriel Ramírez-Vargas, Diana López-Ureña, Adriana Badilla, Josué Orozco-Aguilar, Tatiana Murillo, Priscilla Rojas, Thomas Riedel, Jörg Overmann, Gabriel González, Esteban Chaves-Olarte, Carlos Quesada-Gómez, César Rodríguez

**Affiliations:** 1 Research Center for Tropical Diseases (CIET) and Faculty of Microbiology, University of Costa Rica, San José, Costa Rica; 2 Laboratory for Biological Assays (LEBi), University of Costa Rica, San José, Costa Rica; 3 Leibniz Institute DSMZ-German Collection of Microorganisms and Cell Cultures, Braunschweig, Germany; 4 German Center for Infection Research (DZIF), Partner-site Hannover-Braunschweig, Braunschweig, Germany; 5 Research Center for Zoonosis Control, Hokkaido University, Hokkaido, Japan

## Abstract

The population structure of *Clostridium difficile* currently comprises eight major genomic clades. For the highly divergent C-I clade, only two toxigenic strains have been reported, which lack the *tcdA* and *tcdC* genes and carry a complete locus for the binary toxin (CDT) next to an atypical TcdB monotoxin pathogenicity locus (PaLoc). As part of a routine surveillance of *C. difficile* in stool samples from diarrheic human patients, we discovered three isolates that consistently gave negative results in a PCR-based screening for *tcdC*. Through phenotypic assays, whole-genome sequencing, experiments in cell cultures, and infection biomodels we show that these three isolates (i) escape common laboratory diagnostic procedures, (ii) represent new ribotypes, PFGE-types, and sequence types within the Clade C-I, (iii) carry chromosomal or plasmidal TcdBs that induce classical or variant cytopathic effects (CPE), and (iv) cause different levels of cytotoxicity and hamster mortality rates. These results show that new strains of *C. difficile* can be detected by more refined techniques and raise questions on the origin, evolution, and distribution of the toxin loci of *C. difficile* and the mechanisms by which this emerging pathogen causes disease.

## Introduction

*Clostridium difficile* infections (CDI) are characterized by high morbidity and mortality rates and have been traditionally regarded as nosocomial diseases^1^. Nonetheless, the number of cases of community-acquired CDI is on the rise and recent evidence has revealed that additional reservoirs of *C. difficile* exist^2^. Indeed, pathogenic *C. difficile* strains have been found in livestock and domestic animals as well as in meat^3^. A zoonotic transmission of *C. difficile* has been suggested^4^ and although foodborne CDI has never been confirmed categorically, this species can persist and grow in food products^5^. Furthermore, *C. difficile* has been isolated from water^6^, soil^7^ and other environmental sources^8,9^. Given this variety of origins, it is not surprising that strains of *C. difficile* continue to emerge

The large clostridial toxins TcdA and TcdB have been traditionally regarded as the main virulence factors of *C. difficile*. These glycosyltransferases are encoded in a so-called pathogenicity locus (PaLoc) along with genes that likely influence their synthesis and secretion^10^. Some strains possess an additional toxin with an ADP-ribosyltransferase activity whose coding genes are found at a different chromosomal location in a locus known as CdtLoc^11^. Both the PaLoc and CdtLoc show characteristics of mobile or mobilizable genetic elements (MGEs), including high polymorphism levels and signs of recombination. Accordingly, 34 different toxinotypes^12^ and at least five CdtLoc lineages^11^ have been described until now.

Multilocus sequence typing (MLST) and whole genome sequence (WGS) analyses have revealed that the population structure of *C. difficile* comprises eight clades^9,13^. For many years, the Clade C-I only included isolates from the non-toxigenic sequence types ST-177, ST-178, ST-179, ST-180, and ST-181^14^, yet in 2015 Monot and collaborators described two strains from this clade with atypical monotoxin PaLoc structures^15^.

As part of a routine surveillance of *C. difficile* in TcdB^+^ stool samples from diarrheic patients, we identified three isolates, termed HMX-149, HMX-152, and HSJD-312, that consistently gave negative results in a PCR-based screening for *tcdC*. This unusual finding motivated us to thoroughly characterize them using phenotypic tests, whole-genome sequencing, protein analyses, and experiments in cell cultures and infection biomodels to confirm whether they belong to the Clade C-I and to gain insight into their biology and pathogenicity.

## Results

### Toxin detection tests

**Figure 1:**
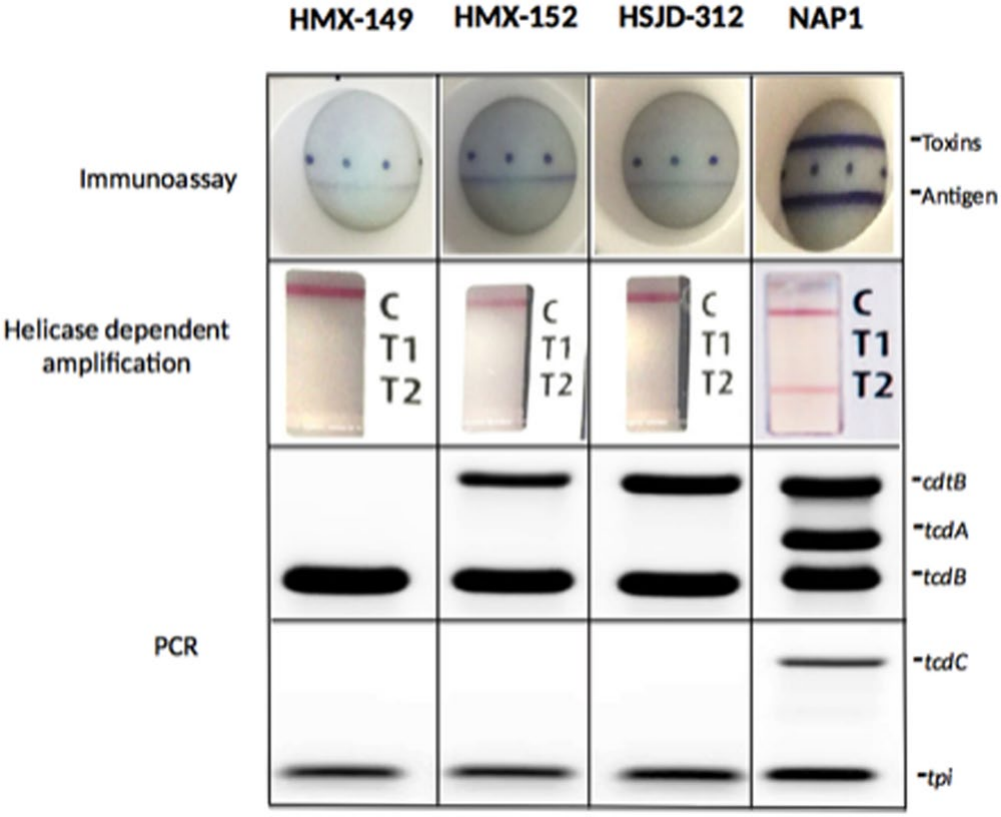
Toxin detection for isolates HMX-149, HMX-152, and HSJD-312. Two rapid tests commonly used in clinical laboratories failed to detect their toxins (upper panel) or toxin genes (middle panel). A PCR-approach, instead, confirmed their identification as *C. difficile* (*tpi*^+^) and revealed that they carry *tcdB* and in two cases *cdtB* (lower panel). The three isolates are negative for *tcdC*. A control NAP1 strain was tested for comparative purposes. Two multiplex PCR reactions were performed, hence two agarose gels appear in the figure. The first reaction targets *cdtB*, *tcdA*, and *tdc*. The second amplifies *tcdC* and *tpi*.

Isolates HMX-149, HMX-152, and HSJD-312 gave negative and weak positive results in a rapid test for immunological detection of TcdA/B and the GDH antigen, respectively, (Fig. 1, upper panel) and were negative for *tcdA* in a helicase-dependent amplification test (Fig. 1, middle panel). However, by PCR we determined that the three isolates indeed carry *tcdB* and that two of them, HMX-152 and HSJD-312, have *cdtB* (Fig. 1, lower panel). The genes *tcdA* and *tcdC* were not detected in any of the three isolates, suggesting that their PaLocs are atypical (Fig. 1, lower panel).

### *In silico* confirmation of toxin loci

**Figure 2:**
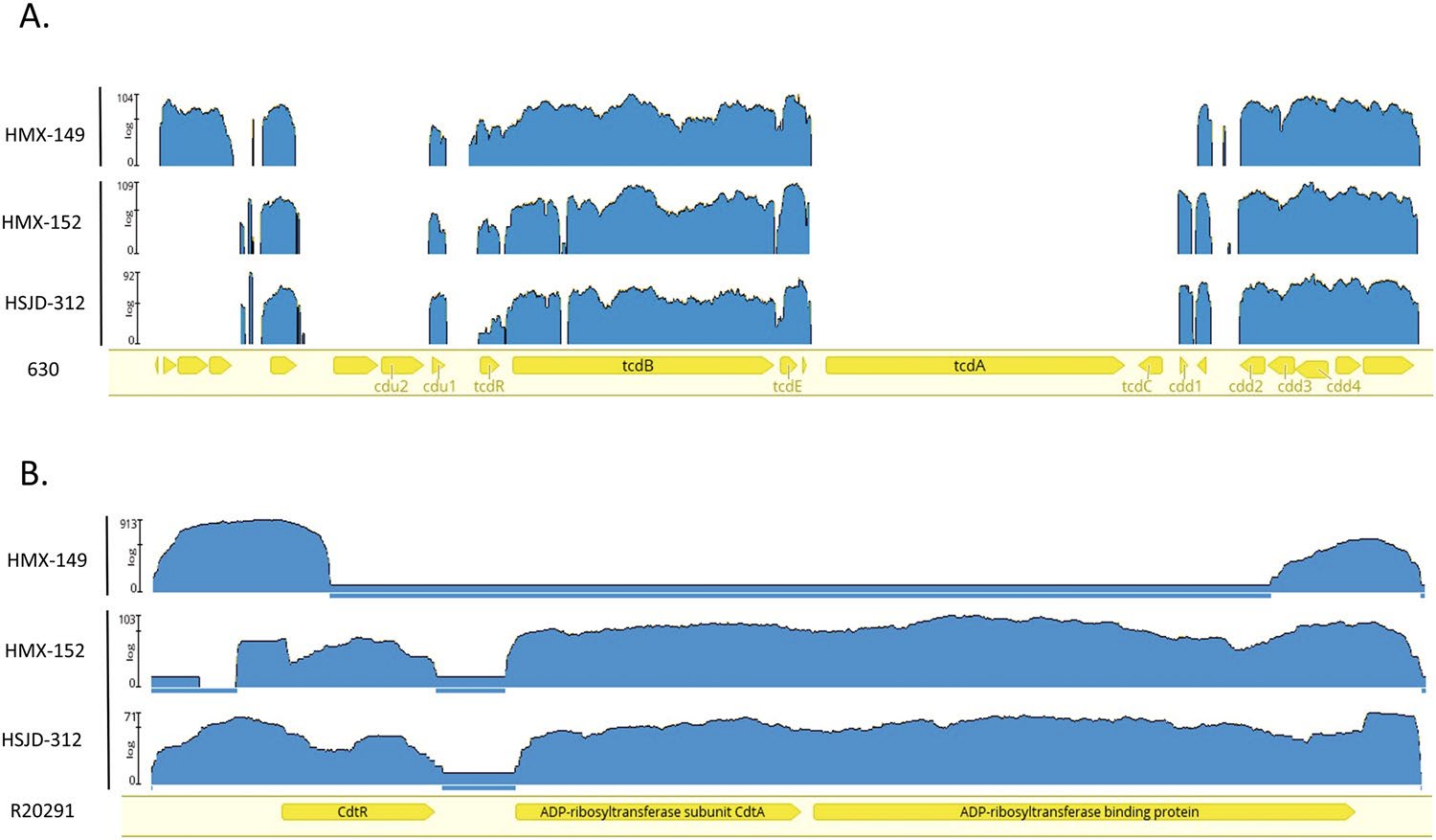
Mapping of NGS reads from isolates HMX-149, HMX-152, and HSJD-312 to PaLoc (upper panel) and Cdtloc (lower panel) sequences from reference strains. These three isolates lack reads annealing to the *tcdA* and *tcdC* genes of strain 630. In addition, the reads obtained for isolate HMX-149 do not map against the CdtLoc of strain R20291.

Mapping Illumina reads to the genome of the reference strain 630 confirmed that HMX-149, HMX-152, and HSJD-312 lack *tcdA* and *tcdC* (Fig. 2A) and revealed that the PaLoc subtypes in HMX-152 and HSJD-312 are closely related. HMX-149, instead, has a different PaLoc structure and integration site (Fig. 2A).

Mapping also demonstrated that HMX-152 and HSJD-312 carry full *cdtR*, *cdtA*, and *cdtB* alleles (Fig. 2B). By contrast, the reads of isolate HMX-149 only mapped to the 5′ end of *cdtR* and to the 3′ end of *cdtB* in the genome of reference strain R20291 (Fig. 2B), confirming that it lacks the binary toxin.

### Strain typing and classification

**Figure 3:**
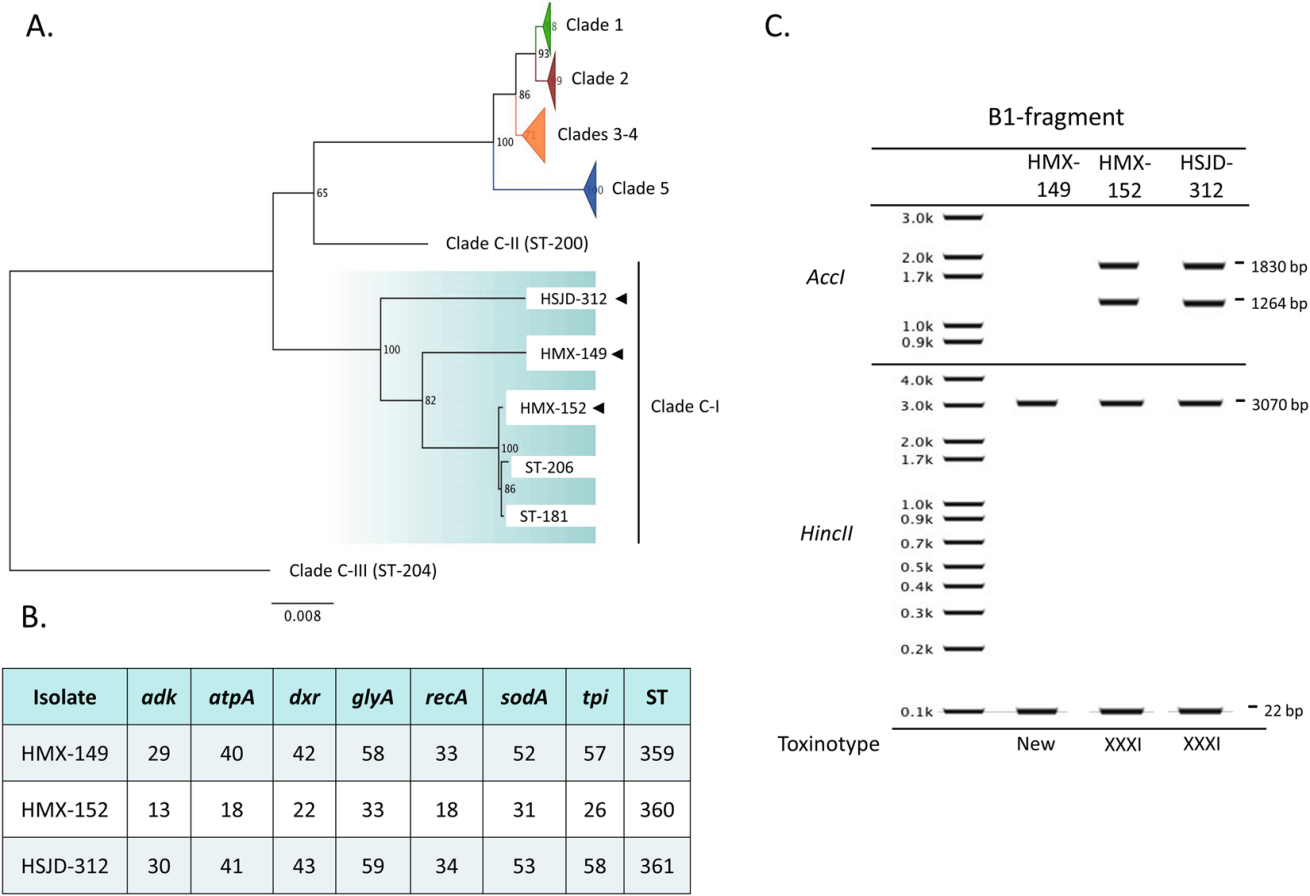
Isolate typing. As determined by wgMLST, isolates HMX-149, HMX-152, and HSJD-312 belong to the Clade C-I (**A**) and carry novel allele combinations (**B**). Through digestion of the B1 fragment of *tcdB* (**C**), isolate HMX-149 was found to represent a new toxinotype and HMX-152 and HSJD-312 were classified as toxinotype XXXI.

We obtained low digital DNA-DNA hybridization percentages (43–44.5%) when we compared draft WGS from our isolates to the genomes of the reference strains 630 (Clade 1), R20291 (Clade 2), M68 (Clade 4), and M120 (Clade 5), confirming that our *tcdC*^−^ isolates belong to a highly divergent *C. difficile* clade. Correspondingly, MLST analyses confirmed that HMX-149, HMX-152, and HSJD-312 are closely related to the two known sequence types (STs) of toxigenic clade C-I strains (ST-181 and ST-206, Fig. 3A). Within this clade, HSJD-312 was more closely related to ST-181/206 isolates than to HMX-149 and HMX-152 (Fig. 3A). Our isolates had novel allele combinations and were assigned to the STs 359, 360, and 361 in the PubMLST *C. difficile* database (Fig. 3B).

The ribotypes obtained for isolates HMX-149, HMX-152, and HSJD-312 are new in the Canada NML database (NML210, NML211, NML212). Moreover, isolates HMX-149 and HSJD-312 were linked to unseen PFGE macrorestriction patterns in the same reference center (NML-1108 and NML-1109, respectively). By *in silico* restriction analysis of the B1 fragment of *tcdB*, we classified HMX-152 and HSJD-312 as toxinotype XXXI and gathered evidence to classify isolate HMX-149 to a new toxinotype (Fig. 3C).

### Experiments with cell lines and biomodels

**Figure 4:**
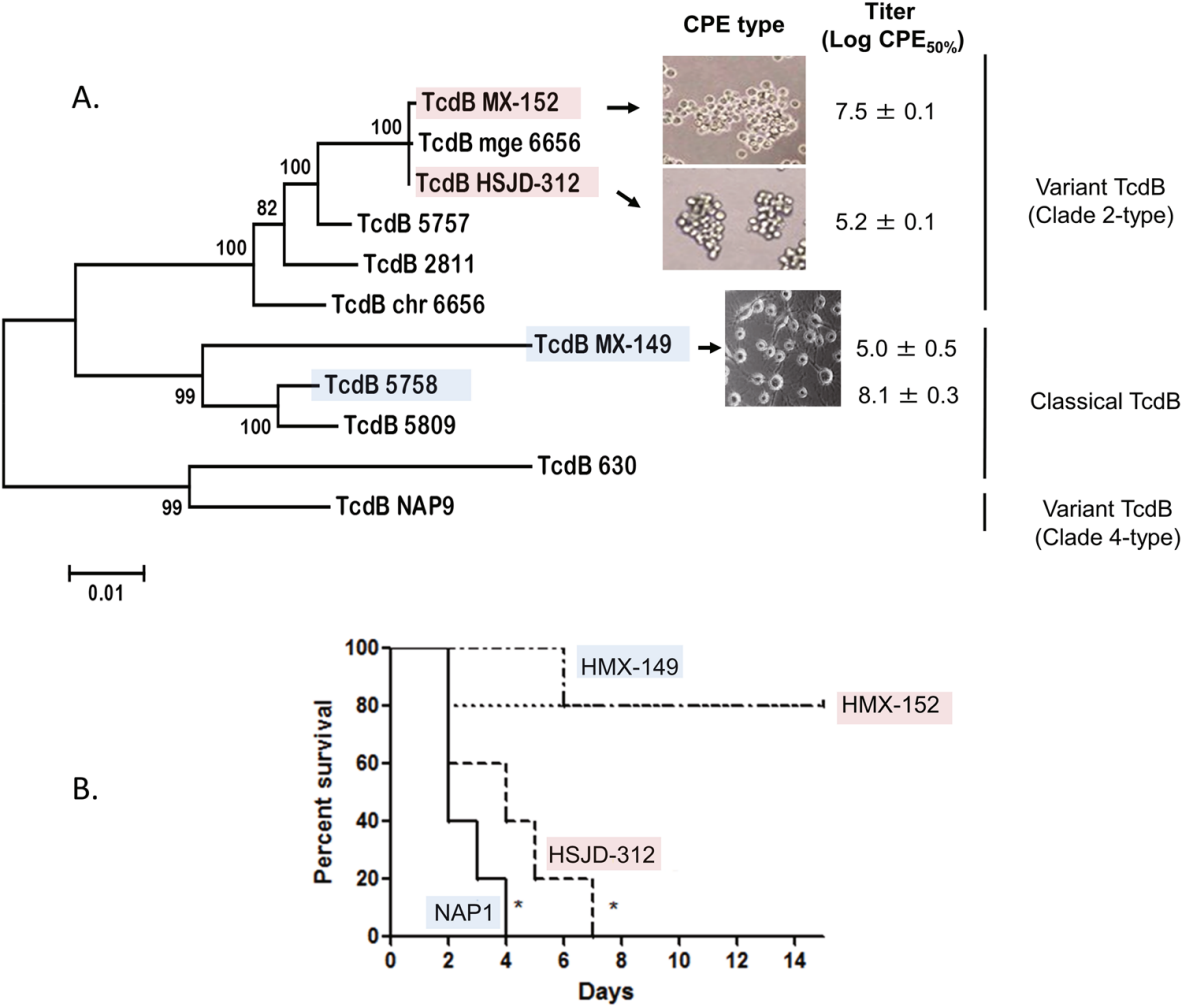
Toxicity to HeLa cells of cell-free supernatants (**a**) and hamster lethal activity (**b**) of isolates HMX-149, HMX-152, and HSJD-312. Isolates HMX-152 and HSJD-312 caused *C. sordellii*-like CPEs with different potencies and have variant TcdB sequences of the Clade 2 type. By contrast, isolate HMX-149 caused an arborizing CPE and its TcdB sequence clustered with classical TcdBs from Clade 2 strains. Isolate HSJD-312 was more lethal than HMX-149 and HMX-152. A control NAP1 strain was tested in parallel. Pink and blue colors were used to highlight variant and classical TcdBs, respectively.

Whereas the supernatants of HMX-152 and HSJD-312 caused *C. sordellii-*like cytopathic effects (CPE) on HeLa cells, HMX-149 supernatants elicited the arborizing CPE seen for a control NAP1 strain (Fig. 4A).

HMX-152 and a control NAP1 strain showed similar cytotoxicity titers (Fig. 4A), as opposed to HMX-149 and HSJD-312, for which we determined much lower titers (Fig. 4A).

All hamsters inoculated with spores from the control NAP1 strain or HSJD-312 died in 4 or 7 days, respectively (Fig. 4B). By contrast, HMX-149 and HMX-152 only induced 20% of mortality (Fig. 4B).

### TcdB features

The predicted TcdB sequences of HMX-152 and HSJD-312 clustered with variant TcdB (vTcdB) sequences (Fig. 4A). TcdB_HMX-149_, by contrast, was more closely related to classical TcdBs (Fig. 4A).

As opposed to TcdB_HMX152_ and TcdB_HSJD-312_ (amino acid identity (AAI) = 91%), TcdB_HMX-149_ showed a higher overall AAI to the TcdBs of the Clade C-I isolates SA10-050 and C10-165 (94–95%, Supplementary Fig. 1). AAI levels ranging from 92 to 94% were observed when the TcdB sequences of SA10-050, C10-165 and HMX-149 (blue), and HMX-152 and HSJD-312 (red) were compared to the TcdB_R20291_ sequence (Supplementary Fig. 1). In this comparison, the TcdBs of SA10-050, C10-165, and HMX-149 showed notorious differences across all four TcdB domains. By contrast, most differences between the variant TcdBs of HMX-152 and HSJD-312 and the classical TcdB of strain R20291 were restricted to their glycosyltransferase (GT) domains (Supplementary Fig. 1).

**Figure 5:**
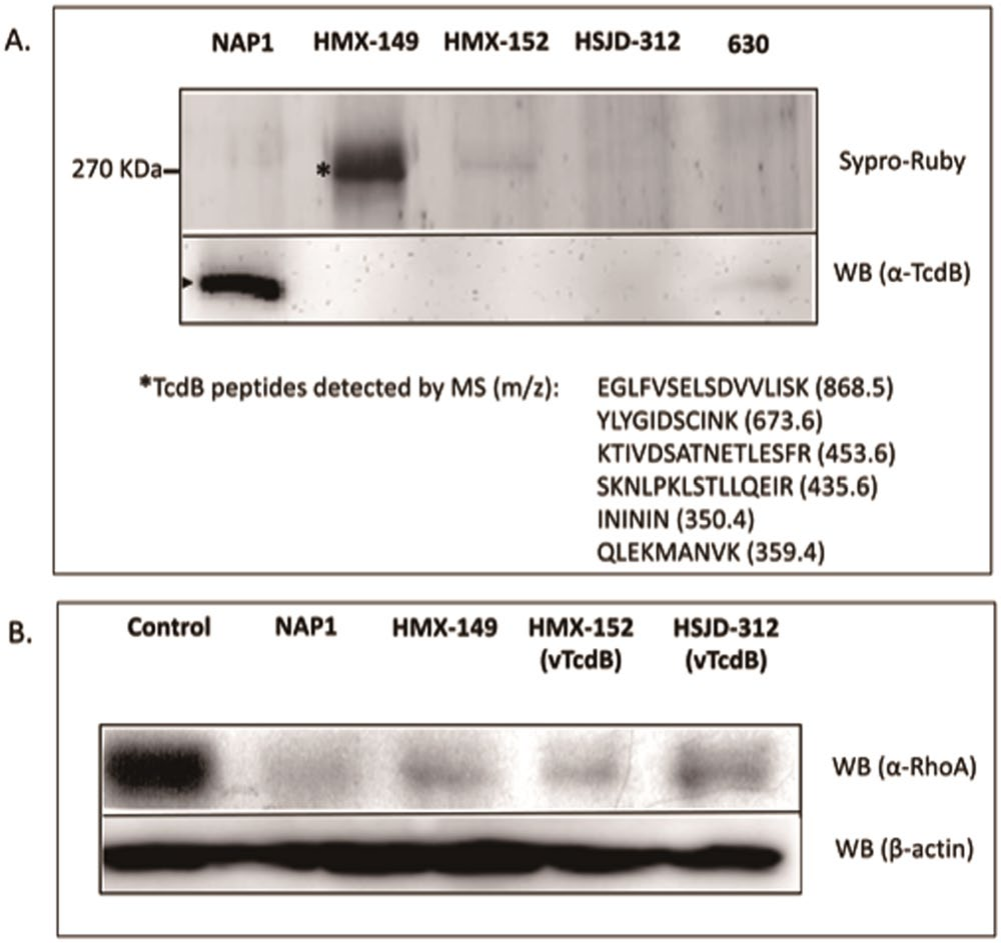
TcdB detection in cell-free supernatants of isolates HMX-149, HMX-152, and HSJD-312 (**a**) and RhoA glycosylation induced by them in HeLa cells (**b**). The TcdBs of HMX-149, HMX-152, and HSJD-312 were not detected by a monoclonal antibody that strongly reacted with the TcdB of the control NAP1 strain tested. Alike a control NAP1 strain, all three Clade C-I isolates glycosylated RhoA (the antibody used does not react with the glycosylated form of RhoA). The 7.5% SDS-PAGE gel in panel A was stained with Sypro Ruby. Only the portion of the gel containing TcdB is shown. Parts of the membranes not reacting with the corresponding antibodies were removed from the western blot (WB) images.

Though HMX-149 showed more TcdB than a hyperproducing NAP1 strain in an SDS-gel (Fig. 5A), none of the TcdBs from our Clade C-I isolates were detected on a Western Blot by a monoclonal antibody that recognizes TcdB_R20291_ and TcdB_630_ (Fig. 5A). RhoA was not detected by an antibody that does not recognize the glycosylated form of this small GTPase in cells exposed to supernatants of HMX-149, HMX-152, and HSJD-312 (Fig. 5B), suggesting that RhoA is a substrate of TcdBs from HMX-149, HMX-152, and HSJD-312 strains.

**Figure 6:**
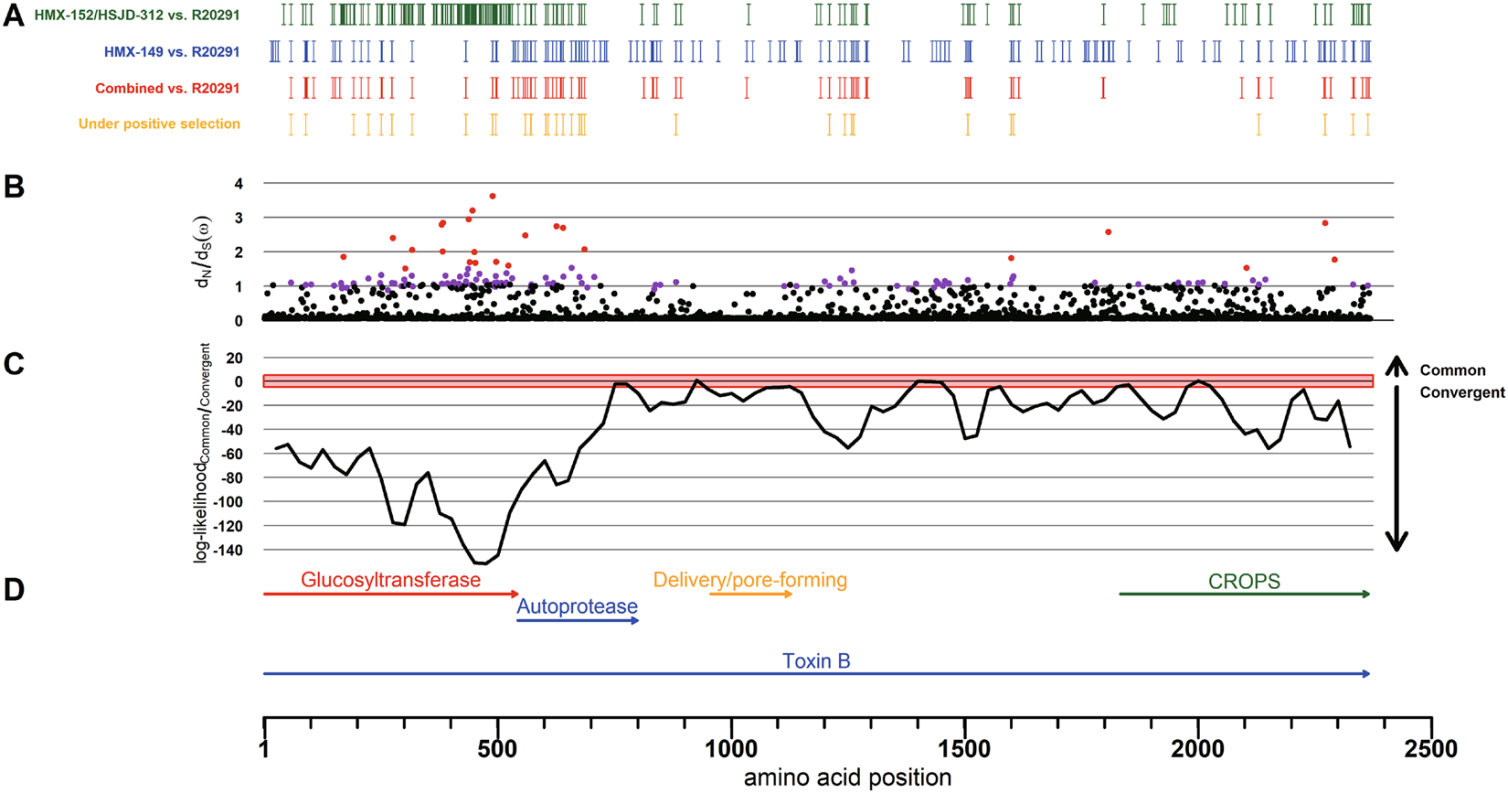
Amino acid differences in TcdB among isolates HMX-149, HMX-152, and HSJD-312 and the reference strain R20291 (**A**): HMX-152 and HSJD-312 (green), HMX-149 (blue), both groups (red). Sites under positive selection are highlighted in yellow. (**B**) *dN*/*dS* ratio (ω). Sites with statistical support of positive selection at *p* < 0.05 and *p* < 0.01 are colored in purple and red, respectively. (**C**) Difference between the log-likelihoods of the common vs. convergent origin hypotheses for the TcdB antibody-escaping feature of the Clade C-I strains. The horizontal axes are adjusted and aligned to the functional annotation shown at the bottom (TcdB_R20291_).

A Bayesian approach to test the hypothesis of a common origin for the antibody non-reactive TcdBs of the Clade C-I isolates and TcdB_R20291_ rendered a negative result (Fig. 6). Instead, it supported the notion that the antibody-escaping feature of TcdB_HMX-149_ and TcdB_HMX-152/HSJD-312_ is a product of convergent evolution. This approach also revealed that most of the amino acid residues under positive selection in these TcdBs are located in their GT domains (Fig. 6).

A comparison of structural models of TcdB_R20291_, TcdB_HMX-149_, and TcdB_HHMX-152/HSJD-312_ in search of epitopes that could explain the antibody-escaping feature of the Clade C-I toxins in the rapid tests did not reveal major variations, except for two subtle differences in the CROPs region (D(2091)N and D(2094)N, Supplementary Fig. 2).

### CdtLoc features

The CdtA and CdtB amino acid sequences of HMX-152, HSJD-312, and the CDT^+^ Clade C-I strains SA10-050 and C10-165 were highly conserved (98–99% AAI). These sequences only showed 71% or 80% identity to the CdtA and CdtB sequences of strain R20291, respectively (Supplementary Fig. 3). These differences were distributed across the entire CdtA sequence or predominantly restricted to the activation and receptor binding domains of CdtB (Supplementary Fig. 3). Relative to CdtA_R20291_, all five Clade C-I CdtA sequences compared share a deletion of six amino acid residues after residue 8, two single residue insertions after residues 28 and 41, and an I309L substitution. A similar analysis using CdtB_R20291_ as reference revealed an insertion of four amino acid residues at position 814 in all Clade C-I isolates as well as a substitution of the C-terminal motif LSVD in strain R20291 by the sequence YEYHK. Moreover, the Clade C-I CdtB sequences analyzed have an asparagine residue at position 209 instead of the classical lysine residue.

### PaLoc structures

**Figure 7:**
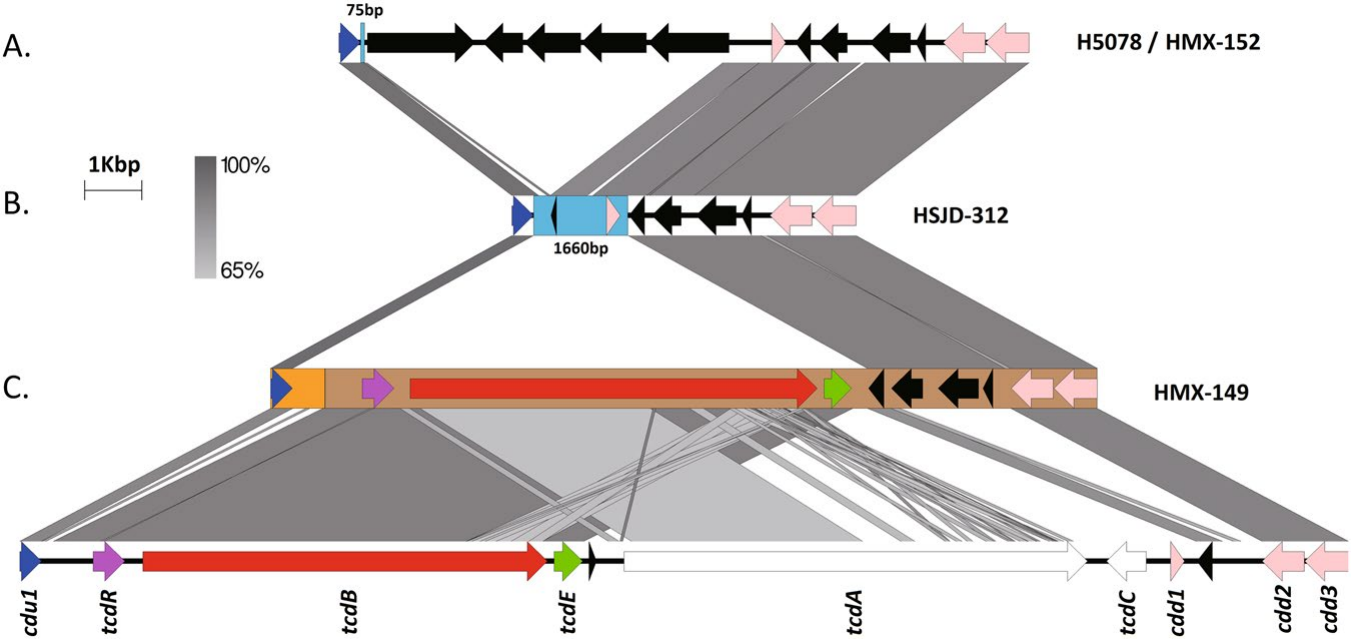
Comparison of the PaLoc insertion site in the chromosome of strain 630 (bottom) and cognate chromosomal regions in the Clade CI isolates HMX-152 (**A**), HSJD-312 (**B**), and HMX-149 (**C**). Despite carrying *cdu1*, *cdd2*, and *cdd3*, only HMX-149 shows an atypical PaLoc at these coordinates.

Although HMX-149, HMX-152, and HSJD-312 have chromosomal *cdu1*, *cdd2*, and *cdd3* genes, only HMX-149 has a monotoxin PaLoc inserted at this location (Fig. 7). Compared to *C. difficile* 630, the chromosomal PaLoc of HMX-149 lacks a small gene for a hypothetical protein, *tcdA*, *tcdC*, and *cdd1*, but shows an insertion of three putative CDS between *tcdE* and *cdd2* (Fig. 7). This insertion was also seen in isolates HMX-152 and HJSD-312 (Fig. 7). Alike the non-toxigenic Clade C-I strain H5078, HMX-152 has five CDS between this sequence stretch and *cdu1* (Fig. 7). We cannot provide information on the chromosomal insertion site of the PaLoc_HMX-149_ because it was found close to a contig end in our draft assembly. On the other hand, when we compared the PaLoc insertion site of our toxinotype XXXI isolates HMX-152 and HSJD-312 with that of the type strain WA151 (Clade 5), we found that the three isolates share a 10 bp sequence by immediately upstream of *tcdR* (GGATGATTTT). These three PaLoc sequences show >90% of identity across *tcdR* and *tcdB*, but start to differ in *tcdE*. The first gap in the alignment appears a few nucleotides before the 3′ end of *tcdE* in WA151, downstream from a AT dinucleotide.

**Figure 8:**
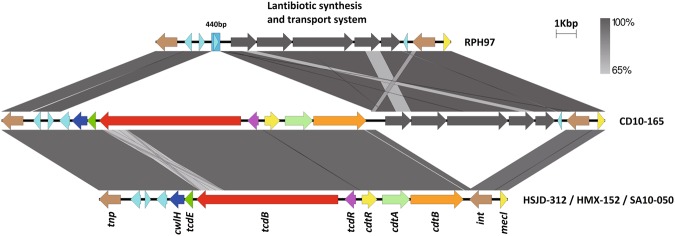
Comparison of the non-chromosomal monotoxin PaLocs of various Clade C-I isolates (middle and bottom). These loci are flanked by a transposase and an integrase. A similar locus lacking toxin genes was found in the non-toxigenic Clade CI isolate RPH97 (top).

The monotoxin PaLocs of HMX-152 and HSJD-312 include *cwlH*, *tcdE*, *tcdB*, and *tcdR* and are preceded by a transposase of the PD-(D/E)XK nuclease family and three genes for hypothetical proteins (Fig. 8). In both strains, we found a full CdtLoc composed of *cdtR*, *cdtA*, and *cdtB* downstream *tcdR*, followed by a gene for an integrase (Fig. 8). HMX-152 and HSJD-312 lack the lantibiotic synthesis and transport system that strain CD10-165 and the non-toxigenic Clade C-I strain RPH97 have upstream of this integrase (Fig. 8).

### PaLoc carriage on putative plasmids

**Figure 9:**
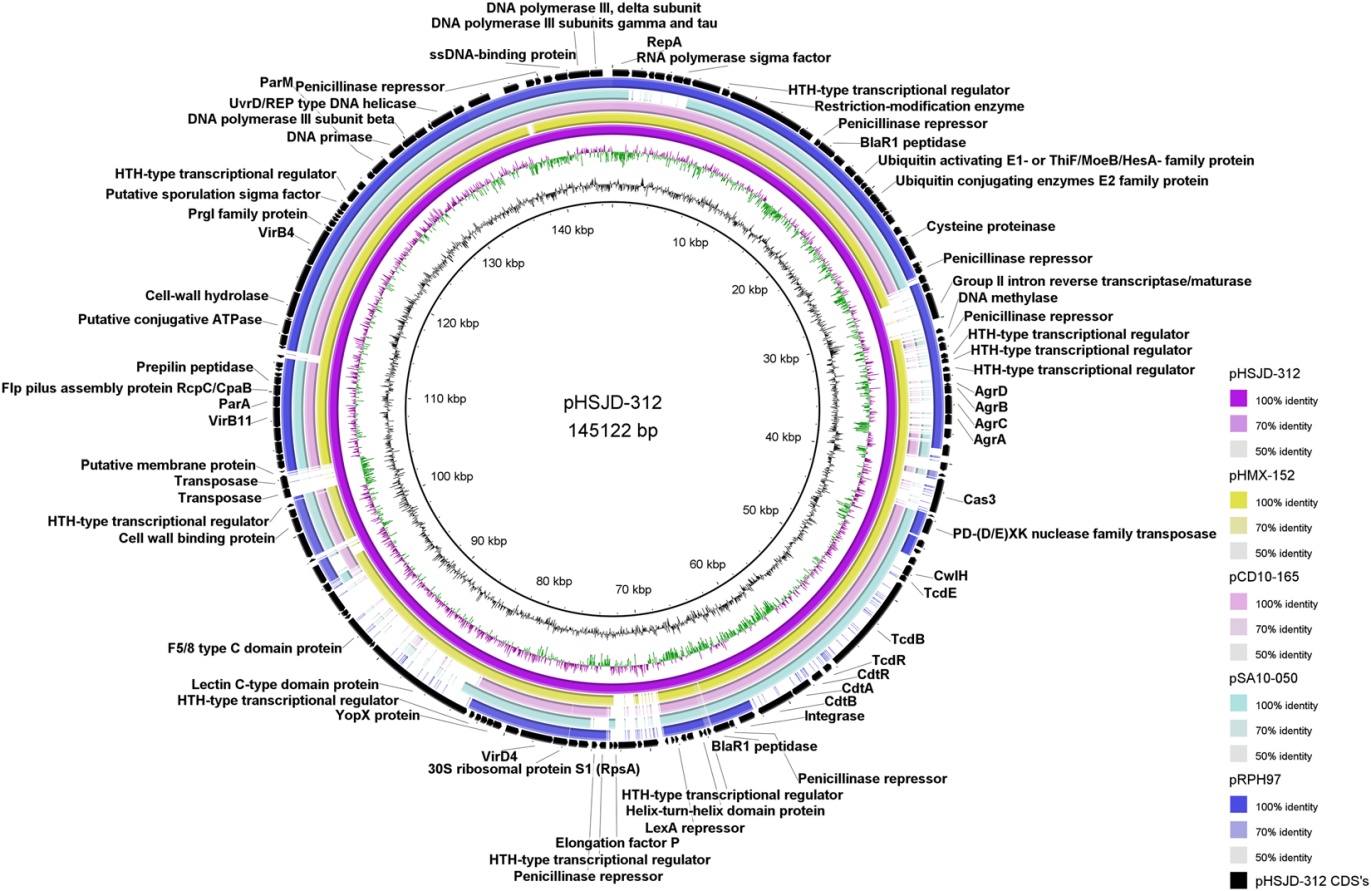
Extrachromosomal circular contigs found in toxigenic and non-toxigenic Clade C-I strains. From inner to outer rings: HSJD-312 (toxigenic, purple), HMX-152 (toxigenic, yellow-brown), CD10-165 (toxigenic, pink ring), SA10-050 (toxigenic, light blue), and RPH97 (non-toxigenic, blue ring). This comparison reveals a differential distribution of toxin loci and the agr locus.

The toxin loci of HMX-152 and HSJD-312 are encoded on circular extrachromosomal molecules. These putative MGEs resemble conjugative plasmids because they have genes for i) a replication initiation protein, ii) proteins of a type IV secretory system, iii) various proteins with AAA-like domains, including a TraE homologue, iv) partition proteins, and v) helicases, a primase, a DNA polymerase III, and DNA binding proteins (Supplementary Table 1 and Fig. 9). IslandViewer4 did not detect genomic islands on the circular contigs of HMX-152 and HSJD-312. Nonetheless, AlienHunter identified their atypical PaLocs, but not their CdtLocs, as a horizontal gene transfer events.

The putative conjugative plasmids of HMX-152 and HSJD-312 also include a full agr locus, a tad-like locus, and putative virulence factors such as a chitinase and cell-wall binding proteins (Supplementary Table 1 and Fig. 9). These molecules are not related to toxin plasmids of *C. perfringens* or *C. sordellii*, including those of strains UMC2, JGS6364 and ATCC9714 (data not shown).

Through *de novo* assembly and read mapping to a closed PacBio sequence of the circular extrachromosomal element of isolate HSJD-312 we confirmed that the Clade C-I strains CD10-165, SA10-050, and RPH97 also carry related circular extrachromosomal molecules. However, they differ through gain or loss of various gene blocks, including the PaLoc, CdtLoc and Agr loci (Fig. 9).

### Antibiotic-susceptibility profiles

Isolates HMX-149, HMX-152, and HSJD-312 were susceptible to clindamycin, vancomycin, rifampicin, and metronidazole (Table 1). They were resistant to ciprofloxacin, but not to moxifloxacin (Table 1).

**Table 1:** Antimicrobial susceptibility of three new toxigenic Clade C-I strains recovered from human patients with CDI.

Strain	Minimum inhibitory concentration (µg/ml)					
	First-line antibiotics for CDI			Antibiotics linked to epidemic potential		
	Clindamycin	Vancomicin	Metronidazole	Ciprofloxacin	Moxifloxacilin	Rifampicin
HMX-149	6	1	1	**>32** ^a^	0.75	3
HMX-152	3	2	0.5	**>32**	1	2
HSJD-312	0.5	1	0.25	**16**	0.75	0.002

^a^Values highlighted in bold represent MIC values above the resistance cutoff.

## Discussion

Here we describe three *C. difficile* strains isolated from human patients and by a detailed polyphasic characterization show that they are unique regarding their classification and toxin loci structures and locations. Moreover, we provide phenotypic data on the pathogenic potential of Clade C-I strains. Altogether, our findings reinforce the notion that the epidemiology of this emerging pathogen is not fully understood and deliver new information on the evolution of the PaLoc and CdtLoc in *C. difficile*. Moreover, since strains HMX-149, HMX-152, and HSJD-312 escape common diagnostic techniques, our findings justify the development of more comprehensive tests for fast and adequate detection of Clade C-I strains in clinics and continuous typing and biological characterization of new isolates to ensure that such strains do not remain unnoticed.

Similar to the Clade C-I strains SA10-050 and CD10-165 from France^15^, our isolates were shown by molecular techniques and NGS to be *tcdA*^−^, *tcdC*^−^, *tcdB*^+^ and to have an endolysin with N-acetylmuramoyl-L-alanine amidase activity next to *tcdE*. We expand this previous knowledge by demonstrating that toxigenic Clade C-I strains may have different TcdB alleles, induce classical or variant CPE types, and cause distinct levels of cytotoxicity and hamster mortalities.

To our surprise, two strains with very similar PaLoc and CdtLoc structures markedly differed with respect to the biological activity of their supernatants (HMX-152 and HSJD-312), raising questions on their pathogenicity mechanisms. Additionally, we demonstrate that toxigenic Clade C-I strains may lack the binary toxin CDT and that CDT carriage did not lead to increased cytotoxicity or lethal activity.

Although we cannot reconstruct the PaLoc acquisition in strains HMX-149, HMX-152, and HSJD-312, their TcdB sequences are related to those of various strains from the Clade 2, which includes R20291 and other epidemic NAP1 strains. We provide indirect evidence that RhoA is glycosylated by them, irrespective of the type of CPE that they induce. This result is intriguing because vTcdBs seldom glycosylate Rho^16^. We cannot explain at this point how TcdB_HMX-152_ and TcdB_HSJD-312_ may target this small monomeric GTPase even though their GT domains notoriously differ from the GT domains of TcdB_R20291_ and other classical TcdBs that typically target RhoA. However, it would be interesting to decipher whether this unexpected ability is related to the positive selection that these GT domains seem to be experiencing. If so, it would be tempting to speculate that vTcdBs are more ancestral and that it has been more advantageous for *C. difficile* to exchange them for TcdBs exerting arborizing CPE and to complement them with TcdA, which has been shown to modulate the activity of TcdB^17^. In support of this hypothesis, some strains of *C. sordellii*, which as *C. difficile* are classified in the Cluster XI of the Clostridia and share biological and immunological traits with *C. difficile*^18^, have toxins in PaLoc-like elements that induce variant CPEs^19^.

To further add controversy to our current understanding of the pathogenesis of CDI, HMX-149 was linked to lower cell cytotoxicity and hamster mortality figures than a NAP1 strain though it noticeably produced more TcdB *in vitro*. This finding does not match the widespread notion that hypervirulence is related to TcdB overproduction^20^, but *in vivo* toxin measurements are required to confirm it.

The structural differences observed among the predicted TcdB 3D structures of strains HMX-149, HMX-152, and HSJD-312 and R20291 were traced back to the residues D(2091)N and D(2094)N, which are located within the contact site of a TcdB neutralizing antibody with therapeutic potential^21^. Based on their divergence from more prevalent *C. difficile* strains, it would be interesting to clarify whether these and other Clade C-I isolates are irresponsive to this type of intervention and therefore require alternative control strategies.

Compared to CDT_R20291_, the binary toxin of toxigenic Clade C-I strains showed marked sequence differences in CdtA and the activation and receptor binding domains of CdtB, as well as deletions and substitutions of single residues and residue blocks. Therefore, it would be relevant to explore whether they are active or show distinct activities. In this regard, the K209D substitution observed in the CdtB sequences of the Clade C-I strains might impair their activation, as this is likely the cleavage site for serine-type proteases^11^.

Based on the reminiscence of the PaLocs of strains HMX-149 and ES130 (TcdA^−^TcdB^+^)^22^ and the sequence shared by HMX-152, HSJD-312 and the toxinotype XXXI strain WA151 upstream of *tcdR*, we postulate the Clade 5 as a possible PaLoc source for some Clade C-I isolates. So far, all toxin genes of *C. difficile* have been found in its chromosome^20^. Surprisingly, the PaLoc and CdtLoc of HMX-152 and HSJD-312 are encoded on a variety of extrachromosomal circular molecules, similar to the LCT toxins TcsL and TcsH of *C. sordellii*^23^. This group of putative plasmids is not restricted to Clade C-I strains, as we recently found another member in a *C. difficile* isolate ST-154 from the Clade 2 (unpublished data). These putative conjugative plasmids show no matches to sequences deposited in public databases, yet based on their annotation we anticipate that they encode additional virulence factors and an *agr* loci, possibly for toxin regulation^24^. This finding confirms the mobility of the CdtLoc, which was recently found in an episomal *C. difficile* bacteriophage^25^.

Besides the mobility that these putative plasmids could confer to their toxin loci, their monotoxin PaLocs were flanked by a transposase and an integrase. This scenario raises questions about the species distribution of TcdB and CDT, as their encoding genes could be present in yet-to-describe hosts. To further sustain this assumption, there are *tcdA*- and *tcdB*- related genes in *C. novyi*, *C. perfringens*, and *C. sordellii* and it has been claimed that the *C. difficile* PaLoc arose by interspecific recombination^13^. Linked to this issue, we urge a systematic examination of the taxonomic position of the Clade C-I of *C. difficile*, as the DNA-DNA hybridization values obtained for our strains were below the 70% threshold commonly used for species delimitation.

As to the antibiotic-susceptibility of strains HMX-149, HMX-152, and HSJD-312, we assayed ciprofloxacin and moxifloxacin because fluoroquinolones (FQ) have been linked to CDI development^26^ and also because FQ-resistance might have facilitated the spread of epidemic *C. difficile* strains^27^. Likewise, rifampicin was tested because it has been postulated as a marker of epidemic strains^28^. The three Clade C-I strains studied are susceptible to these drugs. Nonetheless, we cannot assume that they are not epidemic based on their potential underrepresentation in isolate collections due to the difficulties linked to their detection in clinical laboratories.

Regardless of the severity of CDI induced by HMX-149, HMX-152, and HSJD-312, their isolation from clinical cases along with the unusual conformation and location of their PaLoc suggest that the minimum requirement for the induction of these infections is the ability to secrete at least one toxin targeting small GTPases of the Rho family. Under this scenario, it is expected that *C. difficile* continues its evolution and opens the possibility of the emergence of strains with the ability to cause severe disease in immunocompetent and non-compromised individuals. These concerns call for a continued surveillance on this intriguing pathogen using comprehensive genomic approaches.

## Methods

### *C. difficile* isolation and identification

Stool samples from patients admitted between 11/2011 and 11/2013 to the Costa Rican hospitals San Juan de Dios (HSJD) or Mexico (HMX) that developed antibiotic-associated diarrhea were screened for the presence of *tcdA, tcdB*, and *ctdA/B* using the Xpert *C. difficile* Epi assay (Cepheid). Positive samples were sent to the Laboratory for Anaerobic Bacteriology (LIBA) of the University of Costa Rica, where *C. difficile* was isolated and manipulated in a BSL2 facility. To this end, samples were treated with ethanol to eliminate vegetative cells and thereafter streaked onto CCFA plates (Oxoid). After five days of incubation under an atmosphere of 90% N_2_, 5% H_2_, and 5% CO_2_ in an anaerobic chamber (Bactron, Shellab), yellow ground-glass colonies were subcultured under anaerobic conditions onto Brucella Agar plates supplemented with 5% laked blood, hemin, and vitamin K (BAK). After 2 days, colonies showing yellow-green fluorescence under UV light, positive results in a latex agglutination test (*C. difficile* Test Kit; Oxoid), and typical Gram-staining were identified using a PCR protocol that targets fragments of *tpi, tcdA*, *tcdB*, *tcdC*, and *cdtB*^29^. Once identified as *C. difficile*, three *tcdC*^−^ isolates were cryopreserved at −80 °C in BHI broth (Oxoid) supplemented with 15% glycerol and further examined. We are aware of the fact that *Clostridium difficile* was recently reclassified as *Clostridiodes difficile*^30^. However, we use the former name in this work because the majority of the scientific community continues to do so.

### Rapid tests for toxin detection

Besides the PCR-approach described above for *C. difficile* identification, we used the C. DIFF QUIK CHECK COMPLETE^®^ test (Techlab) for immunological detection of toxins A/B and the glutamate dehydrogenase antigen. Likewise, a helicase-dependent amplification test was used for qualitative detection of *tcdA* (AmpliVue *C. difficile* Assay/Quidel). Both systems were used according to the manufacturer’s instructions.

### Whole genome sequencing and bioinformatic analyses

Genomic DNA was obtained with the DNEasy Isolation Kit (Qiagen) from 48 h cultures of HMX-149, HMX-152, and HSJD-312 in Trypticase soy broth (Oxoid). The quality and yield of these preparations were verified by agarose gel electrophoresis and UV-spectrophotometry using a Nanodrop 2000 (Thermo Fischer Scientific). 2 × 250 bp paired-end reads were obtained through sequencing by synthesis at MicrobesNG (Birmingham, UK). After filtering (-q 30 -p 95) and artifact removal with the Fastx-toolkit (ver 0.0.14), preprocessed reads were assembled using SPAdes (v3.10) or Edena (v3.131028). To resolve the structure of the extrachromosomal circular molecules of HSJD-312 and HMX-152, the genome of HSJD-312 was sequenced on a PacBio *RSII* instrument (Pacific Biosciences) using P6 chemistry. The obtained single-molecule real-time (SMRT) reads were assembled using the “RS_HGAP_Assembly.3 Protocol” included in SMRT-Portal. The plasmidal contig (145122 bp) was circularized and adjusted to start with the *repA* genes. After long-read correction using the “RS_Bridgemapper.1” SMRT-Portal protocol, the plasmid sequence of HSJD-312 was short-read corrected by using Illumina paired-end reads. Prokka (ver 1.11) was used for automated annotation against databases containing *C. difficile* genomes from reference strains. To improve the annotation of the coding sequences (CDS) identified in the plasmid of HSJD-312 we used BLAST, BLASTP, PSIBLAST, BYPASS, PRODOM, SMART, and UniProt searches. Crunch files for comparison of specific loci were generated with WebACT using embl files as input. To determine the structure of the PaLoc and CdtLoc of HMX-149, HMX-152 and HSJD-312, Illumina reads were mapped with Bowtie2 (v.2.3.3.1) and BWA (v. 0.7.16a-r1181) to cognate regions in the reference strains 630 (CP010905.2) and R20291 (FN545816). All genomes and genome comparisons were visualized using Artemis or ACT. Linear and circular comparison figures were prepared with Easyfig and BRIG, in that order. For digital DNA-DNA hybridization, we used the GGDC 2.1 web server (http://ggdc.dsmz.de) and WGS fasta files for strains 630 (CP010905.2), R20291 (FN545816), M68 (N668375.1) and M120 (FN665653.1). Contig files were scanned against the *C. difficile* PubMLST typing scheme using MLST [https://github.com/tseemann/mlst]. The toxin contigs of HMX-152 and HSJD-312 were analyzed with IslandViewer4 for prediction of genomic islands and with AlienHunter for identification of lateral gene transfer events through the implementation of interpolated variable order motifs. Furthermore, we used MUSCLE^31^ to generate alignments of extracted nucleotide or protein sequences and PhyML^32^ and Figtree to transform the alignments into dendrograms.

### Genotyping

We followed standard operating procedures and used databases from the National Microbiology Laboratory of the Public Health Agency of Canada (NML-PHAC) to assign *Sma*I PFGE macrorestriction patterns and ribotypes to the strains. For *in silico* toxinotyping, we extracted the B1 fragment of *tcdB* with primers B1C/B2N and computationally digested these DNA fragments with *Acc*I and *Hinc*II. The resulting RFLP patterns were interpreted following the instructions specified in the most recent toxinotyping scheme^12^.

### Experiments with cell lines and animals

To determine the virulence and pathogenic potential of HMX-149, HMX-152, and HSJD-312 we intoxicated HeLa cells with culture supernatants and fed hamsters with spore suspensions. To determine the type of cytopathic effect (CPE) elicited by the strains and their cytotoxicity (CPE_50_), we intoxicated confluent HeLa cell monolayers with decimal dilutions of cell-free supernatants that were derived from 24 h cultures in TYT broth and monitored the cells by light microscopy. As controls, we used the non-toxigenic strain ATCC^®^700057 and a NAP1/RT027 strain from our collection. To determine the lethal activity of the Clade C-I strains, 10 000 spores resuspended in Dulbecco’s modified Eagle medium (Sigma) were fed to groups of 5 Golden Syrian hamsters (150 to 180 g) that received 30 mg/kg clindamycin on day -2 through the oral route. Hamsters were monitored at 12 h intervals for signs of *C. difficile* infection or death. On days 1, 6, and 12, fecal pellets and intestinal contents of dead and surviving animals were processed for *C. difficile* isolation, and the resulting isolates were typed to confirm the identity of the inoculated strain. A NAP1 strain was tested in parallel for comparison purposes. The Animal Care and Use Committee of the University of Costa Rica authorized this experiment on protocols CICUA 01-12 and CICUA 07-13.

### TcdB detection

Proteins contained in 2 ml of 24 h cultures in TYT broth (3% Bacto tryptose, 2% yeast extract, and 0.1% thioglycolate, pH 6.8) were concentrated using hydroxylated silica particles (StrataClean, Agilent) and thereafter separated by electrophoresis in 7.5% SDS-polyacrylamide gels. These gels were stained with SYPRO^®^ Ruby (ThermoFischer Scientific) or used for electrotransfer of proteins to PVDF membranes (Macherey-Nagel) for Western Blotting with an anti-TcdB monoclonal antibody (2CV, tgcBIOMICS). For signal detection, we used a goat anti-mouse IgG-horseradish peroxidase conjugate (Invitrogen) and the Lumi-Light Plus Western Blotting substrate (Roche).

### Rho glycosylation

Confluent HeLa cells grown with DMEM supplemented with 5% fetal bovine serum were intoxicated with cell-free supernatants from 24 h cultures in TYT. When a CPE was observed, the cells were lysed with 2% SDS and 20 µg of their proteins were resolved SDS-PAGE in 10% gels. The resulting gels were electro-transferred to PVDF membranes (Macherey-Nagel) that were probed with an anti-Rho monoclonal antibody that fails to recognize the glycosylated form of this small GTPase (ab54835, Abcam). Unintoxicated HeLa cells, as well as cells intoxicated with supernatant from a NAP1/027 strain, were assayed as controls.

### TcdB common origin test

To test the hypothesis of a common origin for the Clade C-I TcdBs and TcdB_R20291_ we followed a Bayesian approach that compared the topological hypothesis of common origin versus the null hypothesis of different origin, which implies convergent evolution for the common phenotype. To rule out the possibility of recombination events affecting the analysis, the hypothesis testing was performed in a sliding window approach over the nucleotide multiple sequence alignment of *tcdB* with 150 and 75 nucleotides as window length and step, respectively. In each window, the likelihood of a cluster containing HMX152, HSJD312, HMX149, and R20291, was compared against the likelihood of no clustering. Both likelihoods were estimated with the Stepping Stone method^32,33^ in MrBayes v3.2.6^34^, similar to the recombination analysis. Differences in the loglikelihood >5 were considered as significant evidence in favor of one or the other hypothesis. The hypothesis of positive selection was assessed for all aligned codon sites with MrBayes v3.2.6 by estimating the rate d_N_/d_S_ = ω parameter. The chain was run for 200,000 states, sampling every 100 states and with the average standard deviation of split frequencies <0.01, assuming Nielsen and Yang (NY98) model of codon substitution^35^.

### 3D modeling of TcdB

Tridimensional structural models for the combined repetitive oligopeptides (CROPs) domains of TcdB_HMX-149_, TcdB_HMX-152_, and TcdB_HSJD-312_ were generated with the protein structure homology-modeling server SWISS-MODEL (https://swissmodel.expasy.org) using the structure PDBID 4np4 as template. Models were visualized and colored with PyMol (The PyMOL Molecular Graphics System, ver 1.3).

### Antibiotic-susceptibility profiles

We determined minimum inhibitory concentrations for clindamycin, vancomycin, rifampicin, and metronidazole using BAK plates and E-tests (bioMérieux) according to the manufacturer’s recommendations. For susceptibility categorization, we used the resistance breakpoints recommended by the CLSI in document M100-S27^36^.

## Electronic supplementary material

Supplementary Figures

## Data Availability

Illumina sequencing data and stats are available at https://microbesng.uk/portal/projects/149D9096-03DF-4C6B-BF5F-2119D1AE7B68. The plasmid sequence of HSJD-312 was deposited in Genbank under the accession number MG973074. Other datasets generated and analyzed during this study are available from the corresponding author on reasonable request.
