# Supplementary material for: Novel Clade C-I Clostridium difficile strains escape diagnostic tests, differ in pathogenicity potential and carry toxins on extrachromosomal elements
Source: Sci Rep. 2018 Sep 17;8:13951. doi: 10.1038/s41598-018-32390-6 (PMC6141592; doi:10.1038/s41598-018-32390-6)
Supplement: Supplementary file 1 — Supplementary Figures [file 41598_2018_32390_MOESM1_ESM.pdf]

Supplementary Figures for:

Novel Clade C-I *Clostridium difficile* strains escape diagnostic tests, differ in pathogenicity potential and carry toxins on extrachromosomal elements

Gabriel Ramírez-Vargas[&a], Diana López-Ureña[&a], Adriana Badilla[&a], Josué Orozco-Aguilar[b], Tatiana Murillo[a], Priscilla Rojas[a], Thomas Riedel[c,d], Jörg Overmann[c,d], Gabriel González[e], Esteban Chaves-Olarte[a], Carlos Quesada-Gómez[a], César Rodríguez[a,*]

[a] Research Center for Tropical Diseases (CIET) and Faculty of Microbiology, University of Costa Rica, Costa Rica
[b] Laboratory for Biological Assays (LEBi), University of Costa Rica, Costa Rica
[c] Leibniz Institute DSMZ-German Collection of Microorganisms and Cell Cultures, Germany
[d] German Center for Infection Research, Partner-site Hannover-Braunschweig, Germany
[e] Research Center for Zoonosis Control, Hokkaido University, Japan

[&]These authors contributed equally to this work
[*] Address correspondence to César Rodríguez, Research Center for Tropical Diseases (CIET) and Faculty of Microbiology, University of Costa Rica, San Pedro de Montes de Oca 11501-2060, San José. Costa Rica
Tel: +506 2511 8616
Fax: +506 2225 4384
E-mail: cesar.rodriguezsanchez@ucr.ac.cr

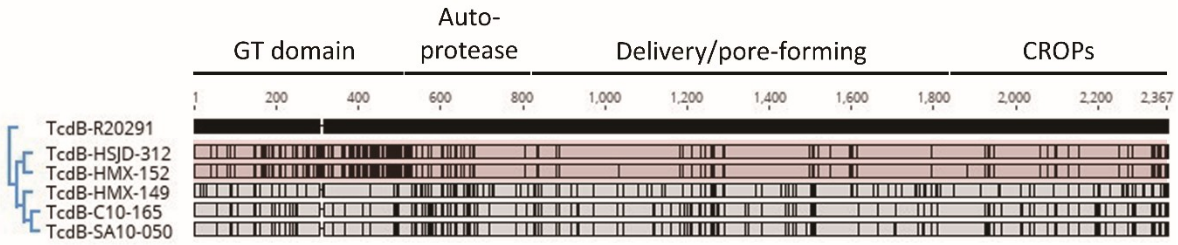

Identity (%)						
**TcdB**	R20291	SA10-050	C10-165	HMX-149	HSJD-312	HMX-152
R20291		94	94	94	92	92
SA10-050	94		100	94	91	91
C10-165	94	100		95	91	91
HMX-149	94	94	95		90	90
HSJD-312	92	91	91	90		100
HMX-152	92	91	91	90	100	

S.Fig 1. Alignment and % identity of predicted TcdB sequences from Clade C-I strains. The TcdB sequence of the Clade 2 strain R20291 was used as a reference.

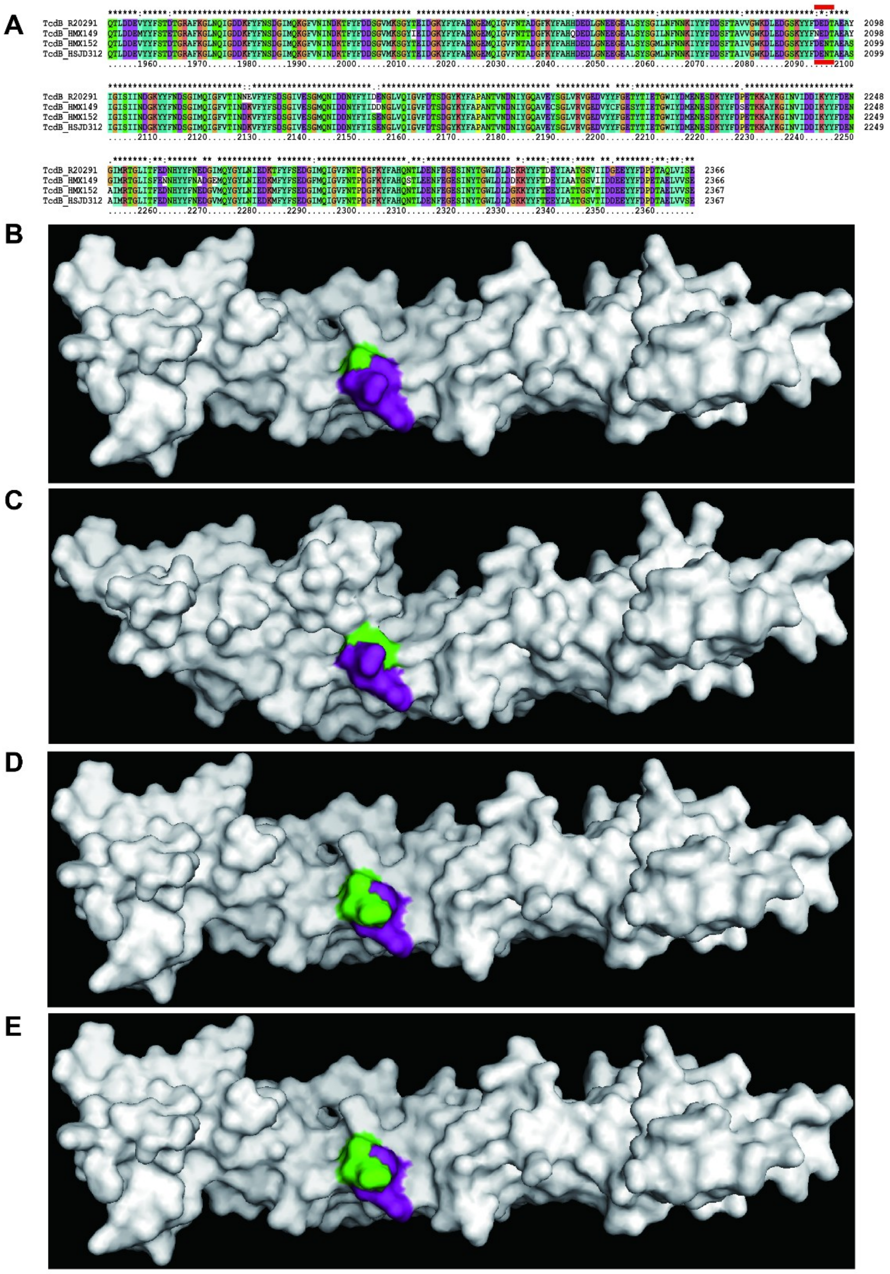

S.Fig 2. Comparison of combined repetitive oligopeptides (CROPs) from strains R20291, MX-149, MX-152 and HSJD-312. A) Multiple sequence alignment of amino acid sequences in the CROPs domain. Alignment positions are shown on the bottom of each line. The motif predicted to be targeted by Bezlotoxumab and evidencing differences among strains is underlined in red. Predicted tridimensional structures for the protein aligned sections were modeled for B) R20291, C) HMX-149, D) HMX-152 and E) HSJD-312. Amino acids underlined in A) are colored in each structure with green for polar uncharged side chains (asparagine and threonine, N and T, respectively) and purple for negatively charged side chains (aspartic and glutamic acid, D and E, respectively)

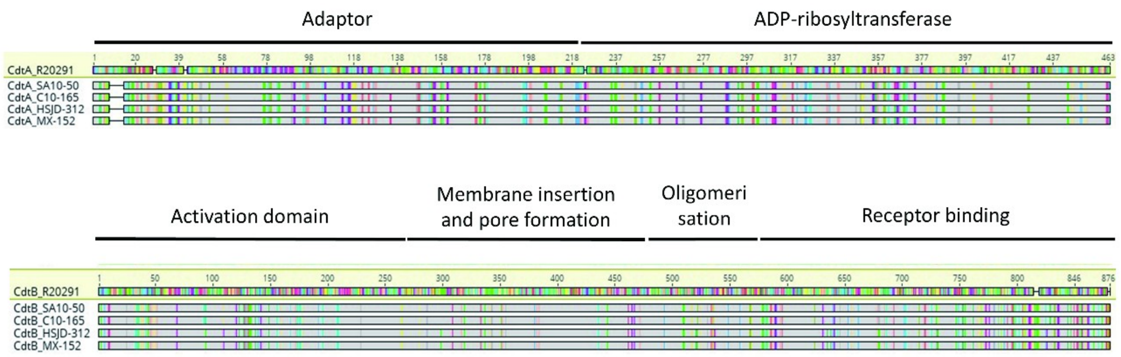

	% identity				
**CdtA**	R20291	SA10-050	C10-165	HSJD-312	HMX-152
R20291		71	71	71	71
SA10-050	71		99	99	99
C10-165	71	99		100	100
HSJD-312	71	99	100		100
HMX-152	71	99	100	100	

	% identity				
**CdtB**	R20291	SA10-050	C10-165	HSJD-312	HMX-152
R20291		81	81	80	80
SA10-050	81		100	98	98
C10-165	81	100		98	98
HSJD-312	80	98	98		100
HMX-152	80	98	98	100	

S.Fig 3. Alignment and % identity of predicted CdtA and CdtB sequences from Clade C-I strains. The CdtA and CdtB sequences of the Clade 2 strain R20291 were used as references.

A

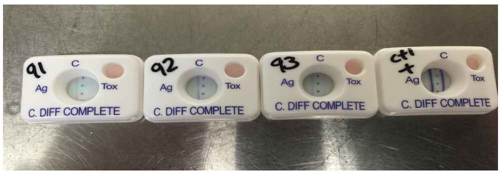

B

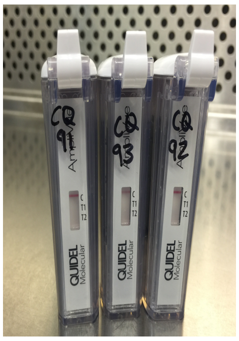

S.Fig 4. Unedited images of the rapid immunochomatrographic test for detection of the GDH antigen and TcdA/B (A) and the molecular test for detection of a *tcdA* fragment (B) shown in Figure 1. (CQ)91: HMX-149; (CQ)92: HSJD-152; (CQ)93: HSJD-312, Ctl+: NAP1 strain

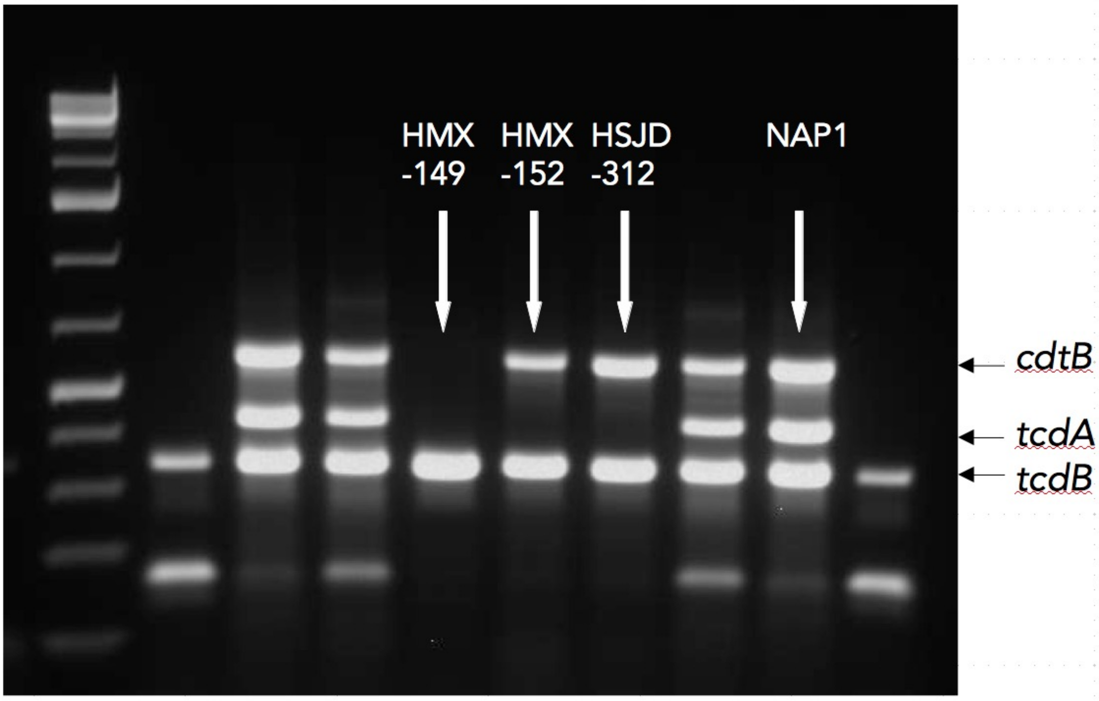

S.Fig 5. Unedited image of an agarose gel loaded with PCR-amplified *cdtB*, *tcdA*, and *tcdB* fragments for HMX-149 (lane 5), HMX-152 (lane 6), HSJD-312 (lane 7), and a control NAP1 strain (lane 9). A 1 kb ladder appears in Lane 1. Only lanes 5-7 and 9 were used in Figure 1.

A

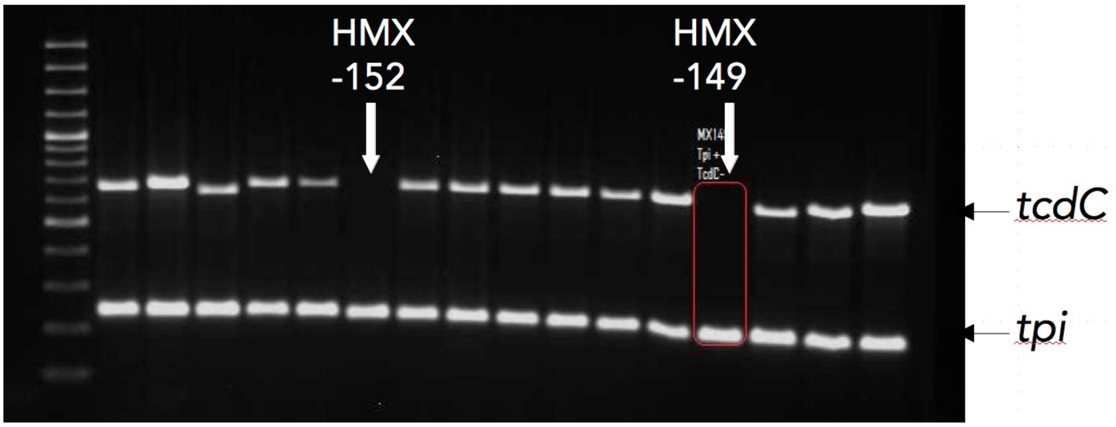

B

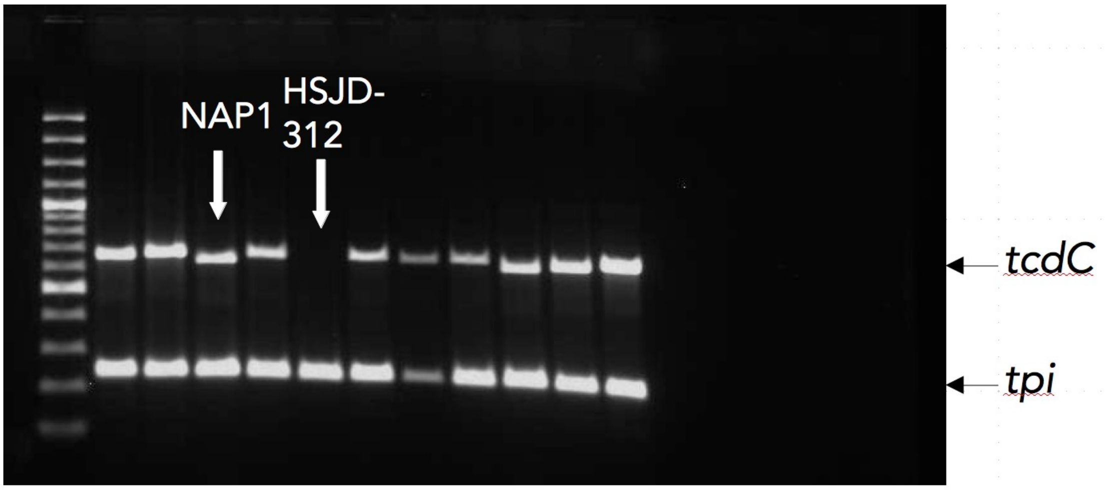

S.Fig 6. Unedited images of agarose gels loaded with PCR-amplified *tcdC* and *tpi* for HMX-152 and HMX-149 (A) or a control NAP1 strain and HSJD-312 (B). A 1 kb ladder appears in the first lane of both gels. Only lanes 7 (HMX-152) and 14 (HMX-149) from panel A and lanes 4 (NAP1) and 6 (HSJD-312) from panel B appear in Figure 1.

A

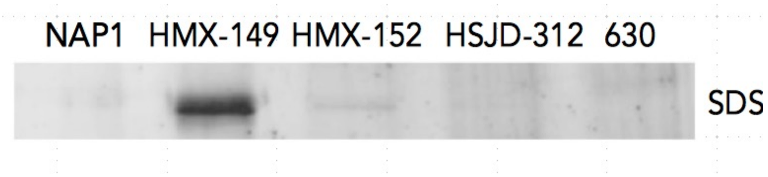

B

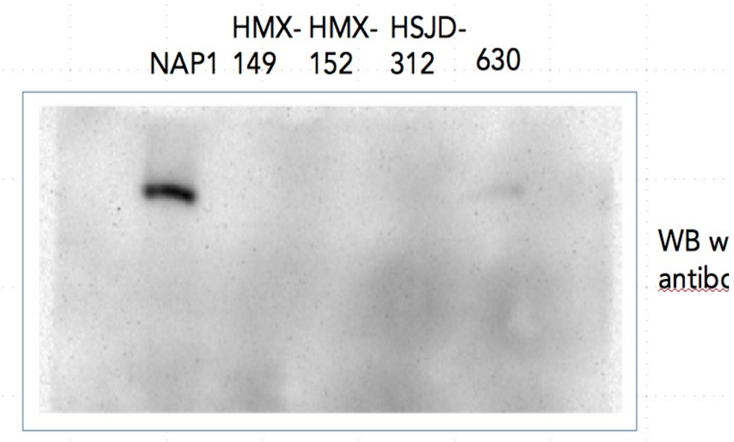

S.Fig 7. Unedited images of the SDS-PAGE (A) transferred to a PVDF membrane (B) for detection of TcdB by Western Blotting in a control NAP1 strain, HMX-149, HMX-152, HSJD-312, and the reference strain 630 (from left to right). These unedited images were used in Figure 5.

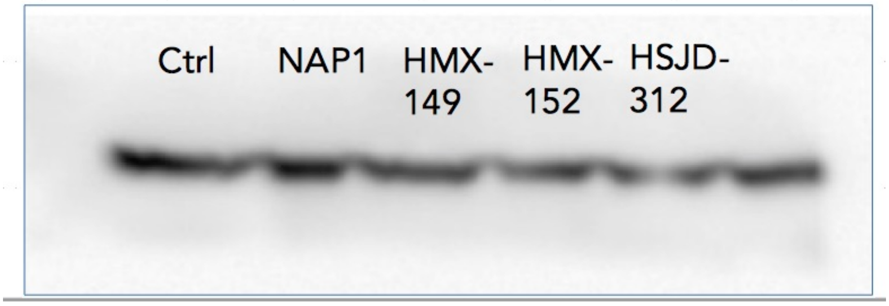

S.Fig 8. Unedited chemiluminescense image of a PVDF membrane probed with an anti-B-actin antibody as load control in the detection of glycosylated RhoA. Lysates from HeLa cells were intoxicated with cell-free supernatants from a NAP1 strain (lane 2), HMX-149 (lane 3), HMX-152 (lane 4), and HSJD-312 (lane 5). Unintoxicated cells were tested in parallel as a control (Ctrl, lane 1). Lanes 1-5 were used in Figure 5. The signal from anti-RhoA antibody is weaker and must therefore be detected using film. The corresponding film was damaged and is not available.
